# Long-term immune profiling of COVID-19 recovered patients: effects of disease severity and vaccination

**DOI:** 10.3389/fimmu.2026.1699992

**Published:** 2026-04-10

**Authors:** Thomas F. Marandu, Lwitiho Sudi, Bernard Mbwele, Abdilahi Kiula, Mohamed Zahir Alimohamed, Pauline Sylvester, Stephen Msangi, Regino Mgaya, Willyhelmina Olomi, Bernard Ngowi, Cecilia Ngatunga, Issakwisa Habakkuk Mwakyula, Mkunde Chachage

**Affiliations:** 1 University of Dar es Salaam-Mbeya College of Health and Allied Sciences, Mbeya, Tanzania; 2 National Institute for Medical Research -Mbeya Medical Research Centre, Mbeya, Tanzania; 3 Department of Biochemistry and Molecular Biology, School of Biomedical Sciences, Muhimbili University of Health and Allied Sciences (MUHAS), Dar es Salaam, Tanzania; 4 Tanzania Human Genetics Organization, Dar es Salaam, Tanzania; 5 Department of Genetics, University Medical Center Groningen, University of Groningen, Groningen, Netherlands; 6 Mbeya Zonal Referral Hospital, Mbeya, Tanzania

**Keywords:** COVID-19 severity, COVID-19 vaccination, HLA alleles, immune profiling, SARS-CoV-2 immunity

## Abstract

**Introduction:**

The COVID-19 pandemic caused varied disease outcomes globally, with individuals experiencing severe, non-severe, or no disease. Immune responses generated post-exposure to SARS-CoV-2 play a critical role in protecting against severe COVID-19 upon re-infection. This study aimed to analyze immune-cell phenotypes and functions in COVID-19 Recovered Patients (C-19RPs) from varying disease severities.

**Objective:**

To compare the immune-cell phenotypes and functions in C-19RPs from different forms of the disease, more than six months post-infection.

**Methods:**

Between September 2021 and July 2023, 101 C-19RPs with hospital data (median age 31) were recruited from Mbeya Zonal Referral Hospital, Tanzania. In addition, seven uninfected and 19 Actively Infected Patients (AIPs) (median age 34 and 58.5, respectively) were included as controls. Blood samples were collected for SARS-CoV-2 serology, immune and genomic analysis, whereas demographic and vaccination data were gathered through a questionnaire.

**Results:**

Serum anti-SARS-CoV-2 levels were similar between severe and non-severe C-19RPs but significantly higher in vaccinated non-severe cases than in unvaccinated ones. Severe C-19RPs and AIPs showed a trend towards decreased switched memory B cells. Frequencies of T-cell subsets were broadly similar across groups, but AIPs had increased central memory and decreased effector memory and effector CD4 T cells. T-cell responses to SARS-CoV-2 nucleocapsid peptides were not affected, but severe C-19RPs had increased CD8 cytokine responses and degranulation upon stimulation with Staphylococcus enterotoxin B (SEB). The frequency of CD56Dim_CD16Bright NK subsets was high in C-19RPs, while CD56Dim_CD16Neg subsets were reduced only in severe C-19RPs. DNA sequence analysis of the HLA from 18 C-19RPs and five uninfected participants revealed 11 and 20 alleles, which were exclusively found in severe and non-severe C-19RPs, respectively.

**Conclusion:**

COVID-19 vaccination was particularly beneficial for non-severe C-19RPs, highlighting the benefits of vaccination in this group. Frequencies of B and NK cell subsets were long-term altered in the C-19RPs, while CD4 T-cell subset alterations were only in the AIPs. The enhanced T-cell response to SEB in the severe C-19RPs suggests potential long-term T-cell hyperresponsiveness, warranting further research. The unique HLA alleles exclusively found in either severe or non-severe C-19RPs may require additional exploration to confirm their association with disease severity.

## Introduction

The COVID-19 pandemic caused varied disease outcomes worldwide, ranging from asymptomatic infections to severe diseases. Despite significant disparities in access to COVID-19 vaccinations, Africa has been the least affected region globally, with 2.5% of the world’s deaths (1). While Africa’s relatively young population may partially account for these observations, biological factors such as sex and genetic background also influence the immune responses of Africans to SARS-CoV-2 (2). Nonetheless, in 2021, Africa was estimated to have one of the highest seroprevalence rates of SARS-CoV-2 in the world (65%), primarily driven by mild infections (3).

It is well established that increased inflammatory cytokines and dysregulation of immune subsets have been associated with the severity of the disease (4, 5). Contrary, non-severe SARS-CoV-2 is characterized by early upregulation of interferon-stimulated genes across different immune cell subsets, reduced cytotoxic potential of T- and Natural Killer (NK) cells, and a monocyte phenotype (4). Host genetic background, such as polymorphisms in HLA genes, influences the immunological outcome of infection in specific study populations (6, 7). Indeed, HLA-DRB1*04:01 is protective of COVID-19 severity in some populations of European ancestry (8).

Given the increasing number of individuals exposed to SARS-CoV-2, there is a growing interest in understanding the state of the immune system post-COVID-19. Kreutmair et al. showed that, unlike other viral acute infections, SARS-CoV-2-associated T and NK immune dysfunction persists 12 weeks after infection, which may play a role in “long COVID” or re-infection risk (4). However, data on the long-term immune effects of SARS-CoV-2 are less described, especially in Africa. Given the high heterogeneity among world populations, understanding the state of immunity maintained after SARS-CoV-2 natural infection is crucial for informing the design of interventions applicable to all populations.

Here, we analyzed the immune phenotypes and functions, and HLA polymorphisms of COVID-19 Recovered Patients (C-19RPs) with different demographic patterns in Mbeya, Tanzania, who had recovered from varying severities of COVID-19. Identifying unique immune and genetic profiles post-COVID-19 may inform on features related to post-acute sequelae of SARS-CoV-2 infection and/or outcomes of re-infection.

## Materials and methods

### Screening, enrollment of participants and sample collection

This cross-sectional analysis was conducted between September 15, 2021, and July 21, 2023, at Mbeya Zonal Referral Hospital (MZRH), with a total of 155 participants recruited from two studies and categorized into three groups. The first group comprised cases, including individuals treated at the MZRH who had recovered from either mild/moderate (hereafter referred to as non-severe) COVID-19 (83) or severe COVID-19 (18). These individuals were recruited after verification in the hospital information system confirmed that they had previously experienced symptoms of COVID-19 and had received treatment at MZRH a month to a year post-diagnosis. The second group consisted of 7 healthy adults with no detectable SARS-CoV-2 antibodies, as determined by both rapid tests (Wantai Biological Pharmacy Enterprise Co., Ltd., Beijing, China) and ELISA (Euroimmun, Lübeck, Germany, EI 2606–9601 G), serving as a negative control. These individuals were randomly screened from a pool of 368 people visiting patients at MZRH. Of the 368 individuals screened, only 35 (9.5%) were negative for SARS-CoV-2 antibodies and were subsequently recruited into the study. However, the absence of anti-SARS-CoV-2 in the blood samples of these 35 participants was further confirmed by ELISA, and only 7 (20%) were true negative samples (**Supplementary Figure 1**). The last group included 19 patients admitted to MZRH who had active severe COVID-19 at the time of recruitment, as determined by clinical assessment and confirmed by viral detection from nasopharyngeal swabs using RT-PCR. COVID-19 disease severity classification was done according to the WHO guidelines (9). A summary chart showing the participant groups and the number of participants included in each respective immunoassay is presented in **Supplementary Figure 1**. For all participants, a total of 17 ml of venous blood was collected in EDTA tubes (BD) for Peripheral Blood Mononuclear Cell and plasma isolation, which were used in subsequent experiments. The sample size was based on the availability of patients and data at that time.

### Anti-SARS-CoV-2 detection by ELISA

Levels of anti-SARS-CoV-2 spike IgG antibodies targeting the recombinant S1 domain of the spike protein in the serum were analyzed by Enzyme-Linked Immunosorbent Assay (ELISA) by using anti-SARS-CoV-2 detection kit (Euroimmun, Lübeck, Germany, EI 2606–9601 G). Samples were processed according to the manufacturer’s instructions. The optical density (OD) values were measured using an ELISA plate reader machine (Tecan, Austria GmbH, Grödig, Austria). Results were reported semi-quantitatively as the OD ratio of the participant sample to that of a calibrator. A sample was considered positive if the ratio was 1.1 or higher. Ratios between 0.8 and 1.1 were classified as borderline.

### Cell staining and flow cytometry

Peripheral blood mononuclear cell (PBMC) isolation was performed using Ficoll-Paque™ Plus medium within 6 hours of blood draw, followed by cell freezing. Thawing of the cells was done for selected samples (**Supplementary Figure 1**) by using a thawing medium according to Horn et al. (10). After thawing, the cells were washed three times with PBS, then counted and divided into three panels. Two panels were designed for T (panel A), B, NK cells, and monocytes (panel B) phenotyping, while one panel (panel C) was used for T cell stimulation and intracellular staining. The list of monoclonal antibodies used in each panel is provided in **Supplementary Table 1A**. Surface staining for panels A and B was done by incubating the cells with the respective antibody master mix for 30 minutes, followed by three washing steps. The cells were then fixed with 1x CellFix solution (BD), prior to acquisition.

For the stimulation panel, cells were restimulated with a pool of SARS-CoV-2 specific nucleocapsid peptides from three SARS-CoV-2 variants (Wuhan, Omicron and Delta) (**Supplementary Table 1B**), each at a final concentration of 1μg/ml (Miltenyi Biotec.), Staphylococcal enterotoxin B (0.6 μg/ml/peptide, Sigma-Aldrich), human cytomegalovirus phosphor protein-65 (pp65) or no peptide (PBS) as a negative control. Stimulation was done overnight (16 to 18 hours) at 37 °C and 5% CO_2_, in the presence of 1x protein transport inhibitor (eBioscience), CD107a-FITC 5μg/ml (Biolegend) and two costimulatory antibodies anti-CD49d (Clone 9F10, BD Pharmingen) and anti-CD28 (Clone CD28.2, BD Pharmingen) (final concentration 1μg/ml each). Following incubation, the cells were washed twice with PBS and then incubated with surface antibodies (**Supplementary Table 1A**) for 30 minutes. Cells were then fixed and permeabilized using Fixation/Permeabilization concentrate and diluent (eBioscience) and stained intracellularly for 30 minutes using intracellular antibodies listed in panel C (**Supplementary Table 1A**). A three-washing cycle with FACS buffer was done before cell acquisition using a CytoFlex Flow cytometer (Beckman Coulter).

Gating analyses were performed using FlowJo™_V10.8.1 (Becton, Dickinson and Company, Ashland, Oregon, USA) and Kaluza version 2.1 (Beckman Coulter Inc., Brea, California, USA) software. Background was removed by subtracting the responding T cell frequencies (by cytokine production or degranulation) in the negative control from those in the corresponding antigen-stimulated samples.

### Quantification of plasma cytokines

Cytokine concentrations in plasma samples from selected individuals with known SARS-CoV-2 exposure status were measured using a multiplex human cytokine assay following the manufacturer’s guidelines (R&D Systems). This assay was designed to detect 14 cytokines: MMP-1, MMP-2, MMP-8, Myeloperoxidase/MPO, S100A8, TNFα, IFNγ, IL-1β IL-8 (CXCL8), IL-12/IL-23 p40, IL17/IL17α, NCAM-1/CD56, CD40 Ligand (TNFSF5), and GM-CSF. Cytokine measurements were performed using a Magpix system and analyzed with xPONENT software version 4.2 (Luminex Corporation, Austin, Texas, USA).

### DNA sequencing and bioinformatics analysis for HLA typing

Genomic DNA was extracted from the collected PBMCs using the QIAamp DNA Mini Kit (Qiagen, Hilden, Germany) following the manufacturer’s protocol. DNA concentration and purity were assessed using a Nanodrop spectrophotometer (Thermo Fisher Scientific), while integrity and accurate quantification were confirmed with the Qubit Fluorometer (Life Technologies). Only samples meeting quality thresholds (A260/A280 ratio ~1.8–2.0 and sufficient DNA yield) were selected for downstream analysis.

Targeted HLA sequencing was performed using a custom-designed AllType NGS panel (One Lambda) on the Illumina MiSeq platform with a paired-end read configuration (2 × 250 bp). The panel was optimized explicitly for high-resolution genotyping of HLA class I (HLA-A, HLA-B, HLA-C) and class II (HLA-DRB1, HLA-DPB1, HLA-DQB1) loci, covering full exons and key intronic regions. Library preparation was performed according to the manufacturer’s protocol, incorporating dual-indexed adapters for sample multiplexing.

Following sequencing, raw reads in FASTQ format underwent initial quality assessment using FastQC (v0.11.9) and MultiQC (v1.13) to evaluate metrics such as read length distribution, base quality scores, GC content, and duplication rates. Adapter trimming was not required, as adapter sequences were effectively removed during demultiplexing, and the sequencing chemistry was optimized to minimize off-target or low-complexity reads. Consequently, high-fidelity, target-specific reads were retained for HLA analysis.

HLA typing was conducted using HLA-HD (v1.7.1), a robust, alignment-based software optimized for high-resolution HLA allele and haplotype inference from NGS data. The tool employs Bowtie2 (v2.5.4) for high-precision mapping of sequencing reads to a curated reference database derived from the IPD-IMGT/HLA database (release version 3.60). HLA-HD implements locus-specific alignment and phasing algorithms to assign alleles at two-field (four-digit) high resolution. It accounts for read depth, allele balance, base quality, and phasing patterns to resolve ambiguous calls. All typings underwent manual curation to confirm accuracy and eliminate artefacts or mismatches. Invalid entries, including unresolved typings, partial (one-field only) calls, or low-confidence assignments, were excluded. A total of 95 unique HLA alleles were identified across the six targeted loci among the 23 sequenced participant samples.

Allelic distributions were stratified by clinical phenotype (severe, non-severe, control), and frequencies were compared across groups. Known HLA-disease associations were cross-referenced with existing literature on SARS-CoV-2 infection, COVID-19 severity, and vaccine responsiveness. Alleles not previously reported in this context were flagged as potentially novel candidates for further investigation.

### Literature and database curation of HLA–COVID-19 associations

To determine whether the HLA alleles identified in our study participants have been previously associated with COVID-19, we conducted a thorough literature and database review using both manual and systematic approaches. We searched PubMed using combinations of keywords such as “HLA,” specific allele names (for example, “HLA-A*02:01”), and COVID-19–related terms including “COVID-19,” “SARS-CoV-2,” “severity,” “susceptibility,” “protection,” and “mortality.” Where relevant, we also included geographic filters such as “Africa” and “sub-Saharan Africa” to help identify studies that included participants from African populations or those of African ancestry. To ensure broad coverage, we additionally searched Google Scholar, medRxiv, and bioRxiv for preprints and grey literature, and manually reviewed article repositories from high-impact journals.

We used several curated databases to cross-check and verify our findings. The HLA-COVID19.org database was our main reference for identifying HLA alleles previously linked to COVID-19 outcomes in the published literature. We used the Allele Frequency Net Database (AFND) to check whether an allele had been previously reported in African populations. However, only studies related to COVID-19 in African populations were used to support the “Reported in Africa” category. Additional verification was done using the Immune Epitope Database (IEDB) for SARS-CoV-2 epitope binding data, and dbMHC (NCBI) for associations with other coronaviruses such as SARS-CoV-1.

All allele names were standardized using the IPD-IMGT/HLA Database. For each of the 95 unique alleles in our dataset, we recorded whether a COVID-19 association had been previously reported, any supporting PubMed IDs globally and for Africa, and the severity of illness in patients from our cohort who carried the allele. We manually reviewed each citation to confirm that the reported association was based on clinical findings and not purely computational predictions.

### Statistical analysis

Data from participants who were actively infected and those who recovered from COVID-19 were extracted from the hospital database and questionnaires. These data were combined with information from laboratory analyses. Statistical analysis was performed using STATA version 16 (Stata Corp., College Station, TX, USA) or GraphPad Prism version 10.4.2 (GraphPad Software, Inc., San Diego, CA, USA). Statistical significance was defined as a p-value less than 0.05.

## Results

### Participants’ demographic and clinical characteristics

The demographic characteristics of 127 participants (**Table 1**) were analyzed of which 73 (57.5%) were males. Participants aged 25–49 years (110; 86.6%) represented the majority compared to those under 24 years (5; 3.9%) and over 50 years (12; 9.5%, p<0.001). Age categories showed a significant relationship with COVID-19 disease severity compared with the control group. Moreover, comorbidities were more common in individuals with active infection. The most frequent comorbidities across all SARS-CoV-2-exposed groups were hypertension and diabetes mellitus type II (**Supplementary Table 2**). About 30% of SARS-CoV-2-exposed participants were vaccinated with either Johnson & Johnson (26, 76.5%) or Pfizer-BioNTech (Comirnaty) (8, 23.5%). The majority (28, 90.3%) of COVID-19 recovered individuals were vaccinated post-infection (Median: 115.5 days (IQR: 75.8-154.3)) while only three were vaccinated before infection (Median: 45 days (IQR: 15-111)). All of the three vaccinated individuals with active infection had received vaccination prior to SARS-CoV-2 diagnosis (Median: 141 days (IQR: 121-162)). Of note, about 21% of COVID-19 recovered participants reported experiencing one or more mild COVID-19-like symptoms at enrolment, including cough, body weakness, and chest and joint pain.

**Table 1:** Participants’ demographic characteristics and related clinical COVID-19 status.

Variable	Number of participants (N = 127)	Non-Severe n (%)	Severe n (%)	Active n (%)	Control n (%)	p-value^#^
Gender						
Male	73 (57.5)	46 (63.0)	12 (16.5)	9 (12.3)	6 (8.2)	0.2789
Female	54 (42.5)	37 (68.5)	6 (11.1)	10 (18.5)	1 (1.85)	
Age group						
<24 yrs	5 (3.9)	2 (40.0)	1 (20.0)	0 (0.0)	2 (40.0)	<0.0001
25–49 yrs	110 (86.6)	81 (73.6)	17 (15.5)	7 (6.3)	5 (4.6)	
>50 yrs	12 (9.5)	0 (0.0)	0 (0.0)	12 (100.0)	0 (0.0)	
Other Comorbidities						
Yes	38(31.9)	19 (22.9)	6 (33.3)	13 (72.2)		0.0003
No	81(68.1)	64 (77.1)	12 (66.7)	5 (22.8)		
Missing	1	0	0	1		
Vaccination status						
Vaccinated	35 (29.7)	28 (33.7)	4 (22.2)	3 (17.6)		0.3145
Unvaccinated	83 (70.3)	55 (66.3)	14 (77.8)	14 (82.4)		
Missing	2	0	0	2		
Type of vaccination (Johnson & Johnson: Pfizer)	26:8	22:6	2:1	2:1		
COVID-19-like symptoms at enrolment						
Yes	21 (20.8)	16 (19.3)	5 (27.8)			0.4205
No	80 (79.2)	67 (80.7)	13 (72.2)			

^#^Statistical test: Chi-square.

### COVID-19 vaccination enhances anti-SARS-CoV-2 serum IgG levels in the non-severe but not the severe group

**Figure 1:**
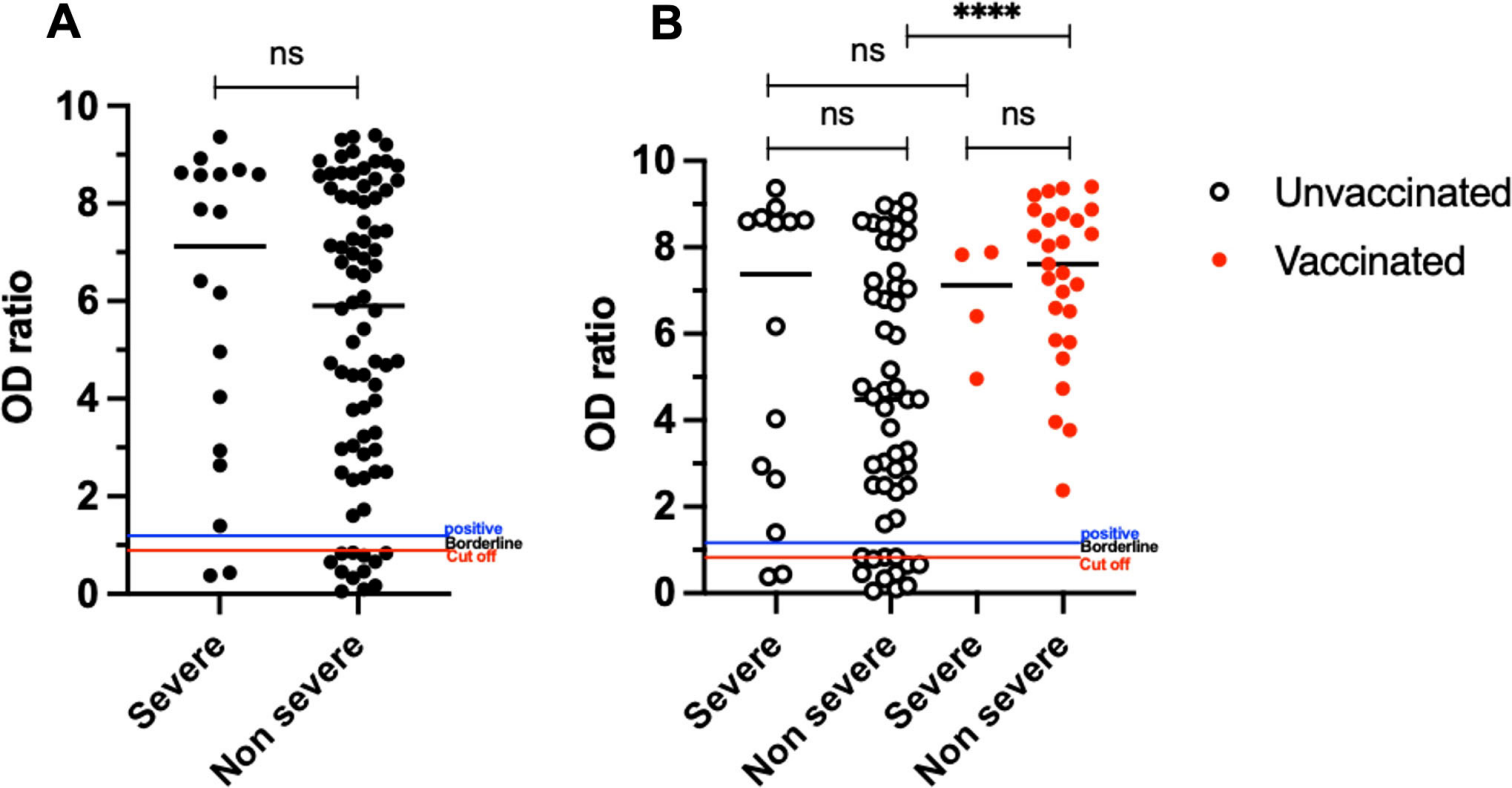
Effects of COVID-19 severity on the anti-SARS-CoV-2 serum IgG levels in recovered patients. Levels of serum anti-SARS-CoV-2 IgG in the severe (n=18) and non-severe (n=80) groups, regardless of vaccination status **(A)**. Levels of serum anti-SARS-CoV-2 IgG in the severe and non-severe C-19RPs who were either vaccinated (open circles) or not (closed red circles) **(B)**. Each dot or circle represents a participant; the median line is indicated. Mann-Whitney U test was used to compare the groups (ns = not significant, ^****^p<0.0001).

To investigate the systemic immune response against SARS-CoV-2, we compared humoral responses and B-cell phenotypes between SARS-CoV-2-exposed (with either active or recovered infection) and controls. The levels of anti-SARS-CoV-2 serum IgG were compared using the Optical Density ratio (OD ratio). The OD ratio between the severe and non-severe groups was comparable (**Figure 1A**). We further discriminated the two groups according to vaccination status; while the median OD ratio remained comparable between severe and non-severe vaccinees, anti-SARS-CoV-2 IgG levels were non-significantly higher in unvaccinated severe than in non-severe C-19RPs (Median: 7.37 vs 4.48, p=0.1599, **Figure 1B**). Interestingly, the OD ratio was significantly higher in the non-severe vaccinated than in the non-severe unvaccinated individuals (Median: 7.61 vs 4.48, p<0.0001). Unvaccinated severe C-19RPs had a comparable serum OD ratio to vaccinated severe C-19RPs.

### COVID-19 affects the B cell compartment in actively infected patients but not in the C-19RPs

**Figure 2:**
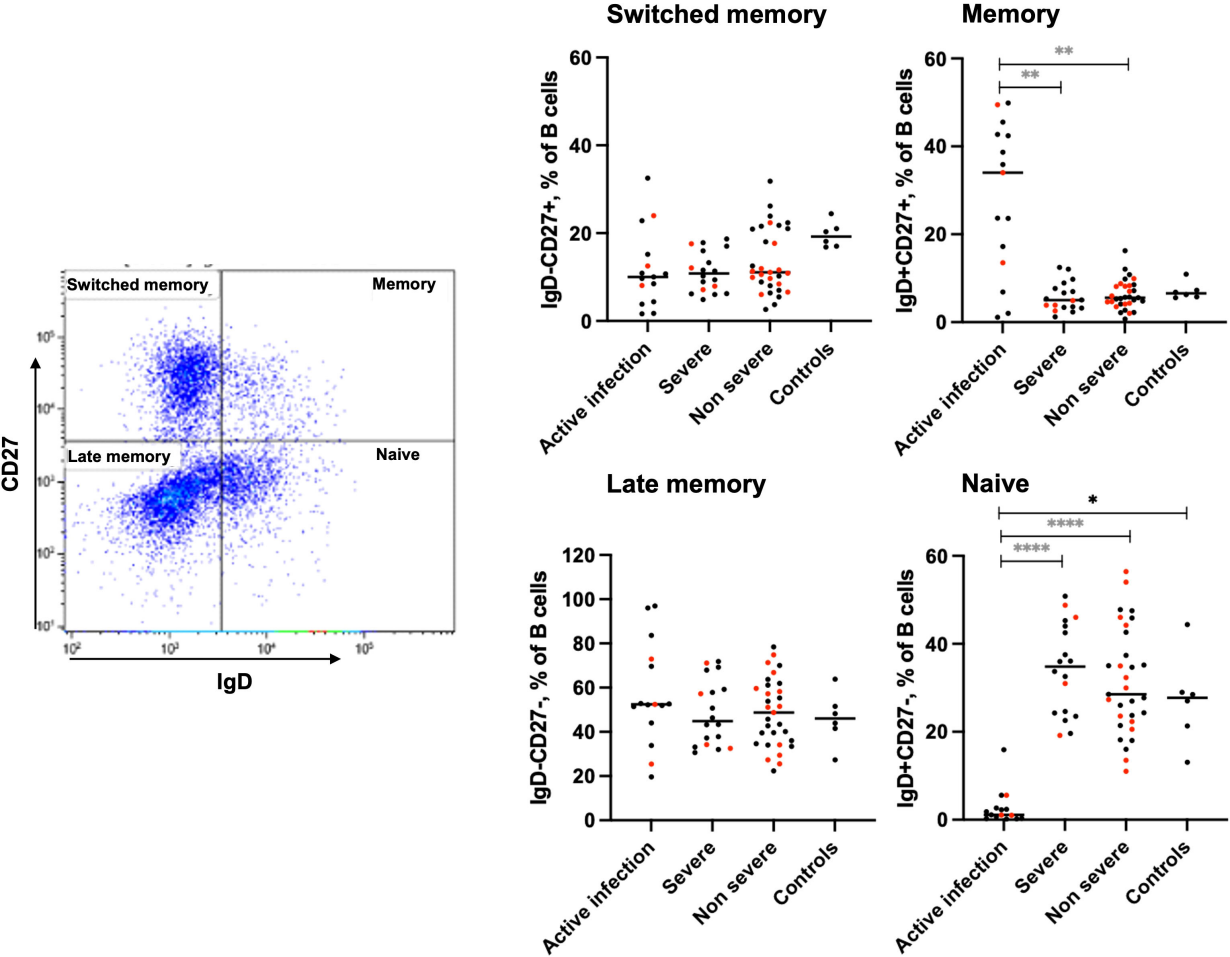
Effects of COVID-19 on the B cell compartment. The frequencies of memory and naïve B cells in the peripheral blood of active infected, severe, and non-severe C-19RPs were compared to uninfected control participants. Each dot represents a participant (red: vaccinated, black: unvaccinated), and the horizontal line represents the median of the group. ^*^p<0.05, ^**^p<0.01, ^****^p<0.0001 (Kruskal-Wallis test followed by Dunn’s post-analysis). A black star indicates a comparison with the control group, while grey stars indicate a comparison with the active infection group.

Three subsets of B cells were analyzed in each of the two groups, and their proportions were compared with those of uninfected controls and active infection. While the three SARS-CoV-2 exposed groups showed similar frequencies of switched memory B cells, there was a non-significant decrease in the fraction of switched memory B cells in the actively infected and severe C-19RPs compared to controls (Median: 10.01, 10.81 vs 19.22%, p=0.060, p=0.0579) respectively. The memory and late memory B cells were not long-term affected by COVID-19 infection when compared to the control group. However, a significant increase in the proportion of memory B cells was observed in individuals with active infection compared to severe (median: 34.03 vs 5.01%, p=0.0007) and non-severe C-19RPs (median: 34.0 vs 5.58%, p=0.0008). The fraction of naïve B cells was also significantly reduced in the active infection group compared to C-19RPs and controls (p<0.0001 (for each group) and p=0.0189, respectively) (**Figure 2**). There was no significant correlation between the serum anti-SARS-CoV-2 IgG OD ratio and the frequency of any of the B cell subsets in both severe and non-severe C-19RPs (**Supplementary Figure 2**).

### Frequencies of CD4 but not CD8 T cell subsets in the actively infected patients are affected but recovered post-COVID-19

**Figure 3:**
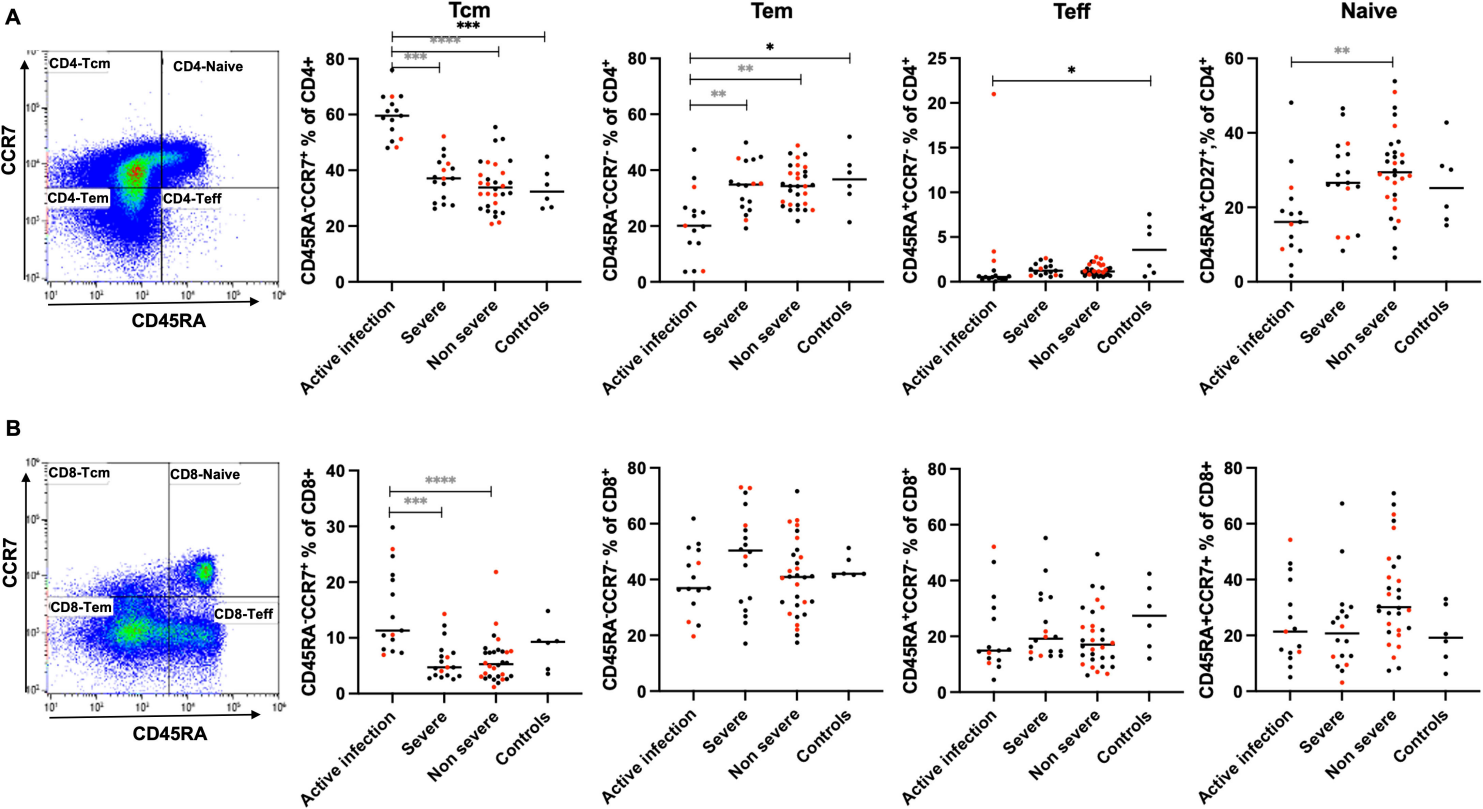
Effects of COVID-19 on the T cell compartment. Frequencies of central memory (Tcm), effector memory (Tem), effector (Teff) and naïve CD4 **(A)** and CD8 **(B)** T cell subsets in the peripheral blood of active infected, severe and non-severe C-19RPs were compared to uninfected control participants. Each dot represents a participant (red: vaccinated, black: unvaccinated), and the horizontal line represents the median of the group. ^*^p<0.05, ^**^p<0.01, ^***^p<0.001, ^****^p<0.0001 (Kruskal-Wallis test followed by Dunn’s post-analysis). Black stars indicate a comparison with the uninfected control group, while grey stars indicate a comparison with the active infection group.

Next, the frequencies of the four T cell subsets (naïve, central memory (Tcm), effector memory (Tem), and terminally differentiated effector cells (Teff)) in the peripheral blood of severe and non-severe C-19RPs groups were compared to those of the control and active infection groups. There was a significant increase in the frequencies of Tcm CD4 T cells in the active infected participants compared to the control group (p=0.0008), but the frequencies of these cells contracted post-COVID-19 recovery in both severe and non-severe groups (p=0.0003, p<0.0001, respectively). In contrast, a significant decrease in Tem and Teff CD4 T cells was observed in the active infection group (p=0.0330, p=0.0194, respectively) compared to the control group, but the frequencies of Tem were restored in COVID-19 recovered patients to levels similar to those in the control group (**Figure 3A**). Interestingly, the frequencies of naïve CD4 T cells were significantly higher only in the non-severe C-19RPs than in the active infection group. The frequencies of Tcm CD8 T cells were significantly lower after COVID-19 recovery in both severe and non-severe groups compared to the active infection group (p=0.0006, p<0.0001). However, for the other CD8 T cell subsets (Tem, Teff, and naïve), a non-statistically significant trend similar to that of the CD4 T cell subsets was observed (**Figure 3B**).

### Degranulation and cytokine T cell responses to SEB stimulation are enhanced in C-19RPs

To assess variability in SARS-CoV-2-specific systemic responses, we measured T cell frequencies in our studied groups following (re)stimulation with a cocktail of pooled SARS-CoV-2 peptides. There were no significant differences between any of the actively infected or C-19RPs and the control groups in CD4 T cell subsets (**Supplementary Figure 3A**). However, a significantly lower frequency of degranulating CD8 T cells was observed in the active infection group compared to the severe C-19RPs (Median: 0.00 vs 0.15% p=0.0199) (**Supplementary Figure 3B**). On the other hand, the frequencies of TNFα responding CD4 and CD8 T cells upon stimulation with pp65 (cytomegalovirus peptide cocktails) were significantly reduced in the active infection compared to the non-severe C-19-RPs (p=0.0050 and p=0.0124, respectively) (**Supplementary Figure 4**).

**Figure 4:**
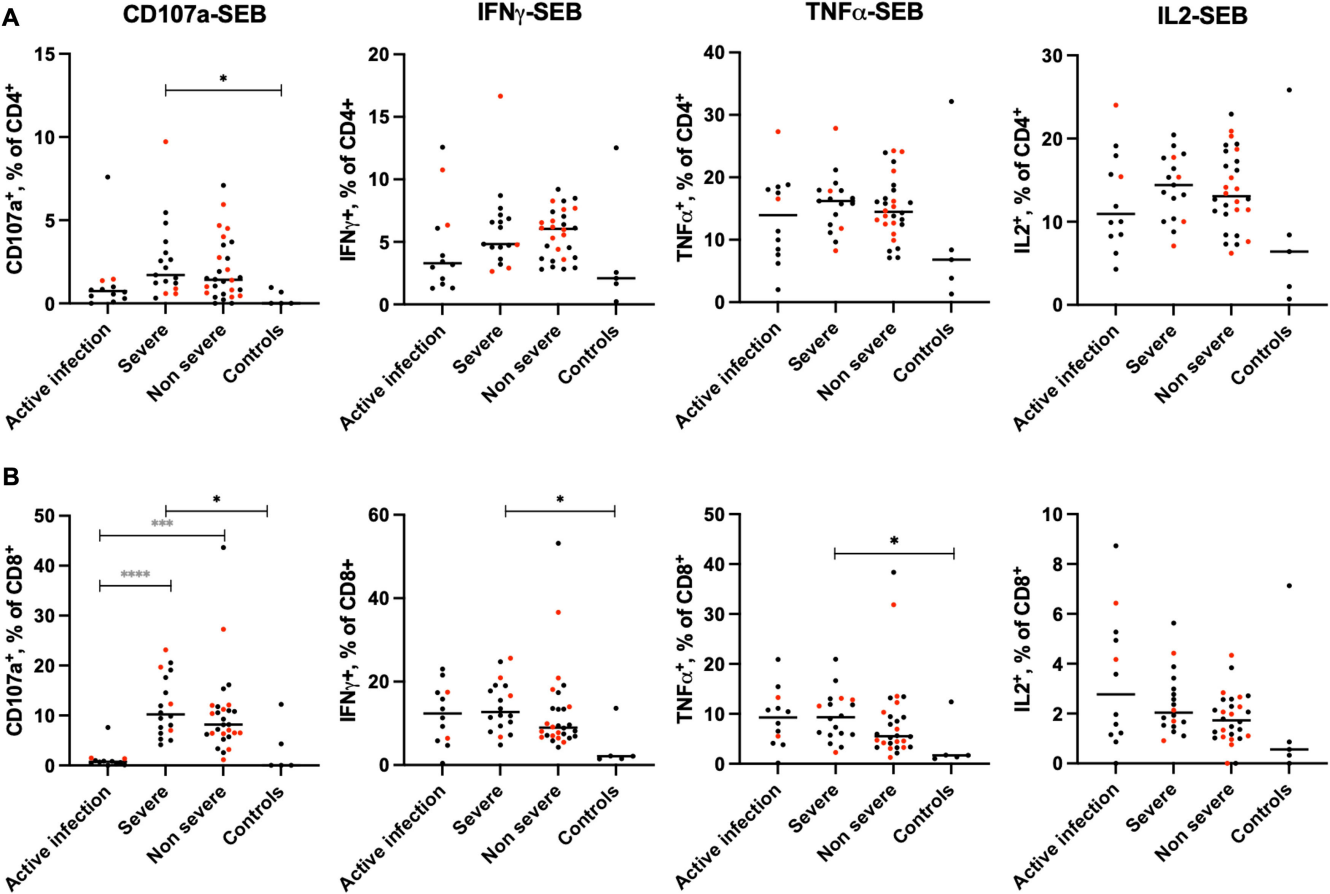
Effects of COVID-19 on the T cell responses to SEB stimulation. The frequencies of CD4 **(A)** and CD8 **(B)** T cell responses to SEB stimulation, measured by degranulation (CD107a) or cytokine production (interferon gamma (IFNγ) or tumor necrosis factor alpha (TNFα)), were compared between the SARS-CoV-2-exposed and control groups. Each dot represents a participant (red: vaccinated, black: unvaccinated), and the horizontal line represents the group median. ^*^p<0.05,^***^p<0.001,^****^p<0.0001 (Kruskal-Wallis test followed by Dunn’s post-analysis). Black stars indicate a comparison with the uninfected control group, while grey stars indicate a comparison with the active infection group.

Noteworthy, frequencies of CD4 and CD8 T cell responses to SEB stimulation by degranulation (CD107a) or cytokine production (interferon gamma (IFNγ), tumor necrosis factor (TNFα) and interleukin-2 (IL2)) were compared between the SARS-CoV-2-exposed and control groups. The frequencies of degranulating CD4 T cells upon SEB stimulation were significantly higher in the severe C-19RPs compared to the control group (median: 1.70 vs 0.00%, p=0.0127), while the frequencies of IFNγ, TNFα and IL2 responding CD4 T cells in active infection, severe and non-severe C-19RPs were comparable to the control groups (**Figure 4A**). The frequencies of degranulating CD8 T cells after SEB stimulation in the active infection group were significantly lower compared to the severe and non-severe C-19RPs (p<0.0001, p=0.0002) (**Figure 4B**). The frequencies of degranulating (CD107a), IFNγ and TNFα responding CD8 T cells were significantly higher in the severe C-19RPs only (p=0.0191, p=0.0131, p=0.0404, respectively) (**Figure 4B**).

When the concentration of cytokines and different inflammatory markers in the serum of active infected participants, severe and non-severe C-19RPs, was compared to the control groups, there were no profound differences that were observed between groups (**Supplementary Figure 5**). However, individuals with active infection had higher levels of inflammatory markers, including TNFα, IL-8, Matrix MetalloProteinase-1 (MMP-1) and -8 (MMP-8), IFNγ, and MyeloPerOxidase (MPO), than severe and/or non-severe C-19RPs (**Supplementary Figure 5**).

### COVID-19 affects the NK cell phenotypes but not monocyte subsets of actively infected patients, severe and non-severe C-19RPs

**Figure 5:**
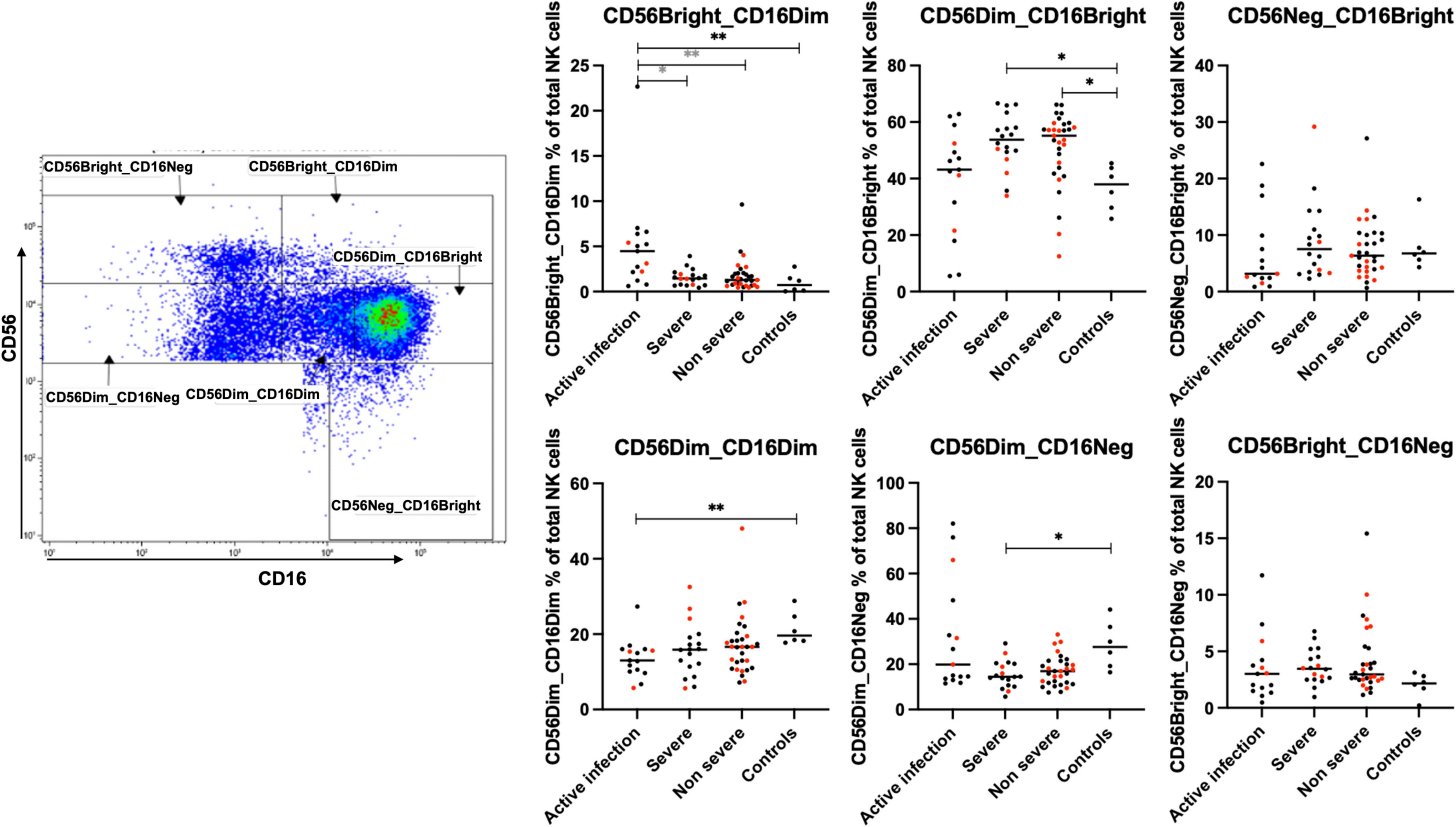
Effects of COVID-19 on the NK cell compartment. Frequencies of the six NK cell subsets in the peripheral blood of active infected, severe, and non-severe C-19RPs were compared to uninfected control participants. Each dot represents a participant (red: vaccinated, black: unvaccinated), and the horizontal line represents the median of the group. ^*^p<0.05,^**^p<0.01 (Kruskal-Wallis test followed by Dunn’s post-analysis). Black stars indicate a comparison with the uninfected control group, while grey stars indicate a comparison with the active infection group.

Six subsets of NK cells were analyzed in each of the studied groups as previously described (11), namely: CD56bright_CD16dim, CD56Dim_CD16Bright, CD56Neg_CD16Bright,CD56Dim_CD16Dim, CD56Dim_CD16Neg, and CD56bright_CD16dim. There was a significantly higher proportion of CD56bright_CD16dim and lower CD56Dim_CD16Dim NK cells in participants with active infection compared to uninfected controls (p=0.0043 and p=0.0075, respectively, **Figure 5**). Of note, the frequencies of CD56bright_CD16dim NK cells were significantly reduced in both severe and non-severe C-19RPs to levels comparable to those in the uninfected controls when compared to the active infection group. The frequencies of CD56Dim_CD16Bright were elevated in both severe and non-severe C-19RPs compared to the controls (p=0.0267 and p=0.0333, respectively). Furthermore, a significant decrease in the frequencies of CD56Dim_CD16Neg NK cells only was observed in the severe C-19RPs compared to the controls (p=0.0319, **Figure 5**).

The frequencies of the three known monocyte subsets in severe and non-severe C-19RPs, were compared with either uninfected controls or actively infected patients. In all three subsets, which are classical monocytes, non-classical monocytes and intermediate monocytes, there were no significant differences between any of the two C-19RPs and the control groups (**Supplementary Figure 6**).

### Association of different HLA polymorphisms with COVID-19 severity

**Figure 6:**
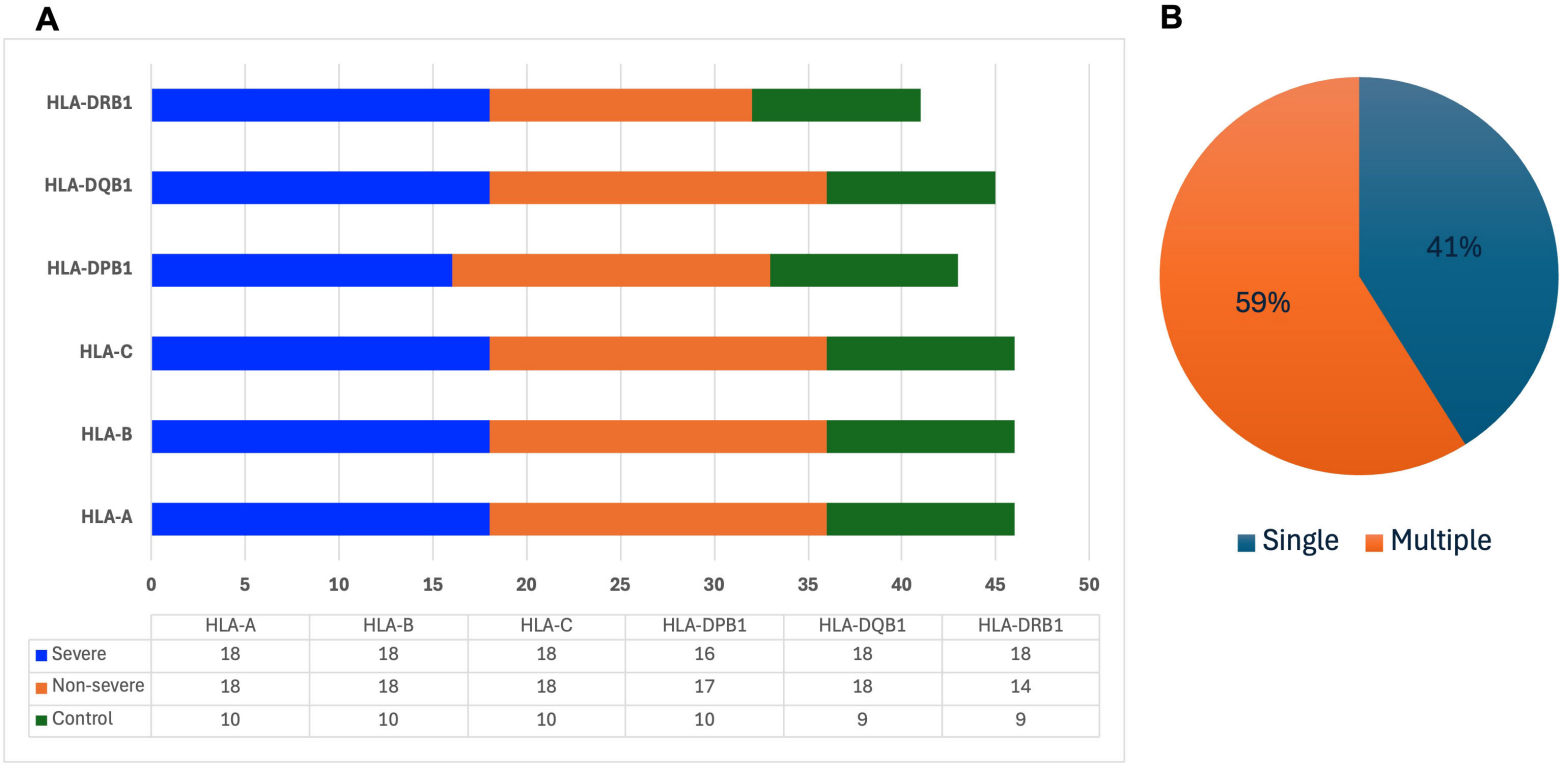
HLA polymorphism analysis. COVID-19 clinical presentation and the distribution of the 267 alleles across the HLA class I and II **(A)**. Proportion of unique alleles expressed by single and multiple participants **(B)**.

High-resolution HLA genotyping in 18 individuals recovered from COVID-19 infection and 5 uninfected controls revealed a total of 267 alleles spanning class I (HLA-A, HLA-B, HLA-C) and class II (HLA-DRB1, HLA-DQB1, HLA-DPB1) loci (**Figure 6A**). Of those, we found 95 unique alleles, of which 39 (41%) appeared only once across the participants, while 56 (59%) appeared in more than one individual (**Figure 6B**).

Among the 9 severe C-19RPs, 11 alleles were identified exclusively in this group (**Table 2**). Of those four alleles have been previously associated with severe COVID-19, while the other seven have not previously been reported in the context of COVID-19 and may represent novel markers of susceptibility in African cohorts.

**Table 2:** Alleles found exclusively in the severe COVID-19 recovered patients.

HLA allele	Reported COVID-19 association?	Notes	Reference
HLA-A*03:01:01	Yes	Associated with increased risk of fever/chills after Pfizer COVID-19 vaccination.	(12)
HLA-A*26:01:01	No	No published association found with COVID-19 severity or susceptibility.	—
HLA-B*08:01:01	Yes	Associated with severe COVID-19 and increased hospitalization risk in Italy.	(13)
HLA-C*14:02:01	No	No known studies link this allele to COVID-19 outcomes.	—
HLA-C*18:03	No	No known studies link this allele to COVID-19 outcomes.	—
HLA-DPB1*124:01	No	No published evidence found regarding COVID-19 association.	—
HLA-DQB1*03:01:01	Yes	Associated with symptomatic COVID-19; increased frequency in severe cases.	(14)
HLA-DRB1*03:91	No	No known studies link this allele to COVID-19 outcomes.	—
HLA-DRB1*12:22	Yes (indirect)	DRB1*12 group is highly prevalent in Africa; possible relevance suggested.	(15)
HLA-DRB1*12:39	No	No published evidence found regarding COVID-19 association.	—
HLA-DRB1*13:12:01	No	No known studies link this allele to COVID-19 outcomes.	—

In the nine non-severe C-19RPs, 20 alleles were found exclusively in this group (**Table 3**). Among these, four alleles had documented protective associations, while no published studies are available to support associations for the remaining 16 alleles in this group.

**Table 3:** Alleles found exclusively in the non-severe COVID-19 recovered patients.

HLA Allele	Reported COVID-19 association?	Notes	Reference
HLA-A*02:02	Yes	Identified as a low-risk allele for susceptibility.	(16)
HLA-A*26:12	No	No published evidence currently available.	—
HLA-B*07:102	No	No published evidence currently available.	—
HLA-B*14:02:01	No	No published evidence currently available.	—
HLA-B*49:01:01	No	No published evidence currently available.	—
HLA-C*03:03:01	No	No published evidence currently available.	—
HLA-C*05:01:01	Yes	Associated with protective effect against severe disease.	(17)
HLA-C*07:01:02	No	No published evidence currently available.	—
HLA-C*07:04:01	No	No published evidence currently available.	—
HLA-C*08:02:01	Yes	Associated with protection from critical disease.	(18)
HLA-C*17:01:01	No	No published evidence currently available.	—
HLA-DPB1*03:01:01	No	No published evidence currently available.	—
HLA-DPB1*17:01	Yes	Associated with low DPB1 expression; possible impact on immune response.	(19)
HLA-DPB1*30:01	No	No published evidence currently available.	—
HLA-DQB1*06:04:01	No	No published evidence currently available.	—
HLA-DQB1*06:09:01	No	No published evidence currently available.	—
HLA-DRB1*03:07	No	No published evidence currently available.	—
HLA-DRB1*10:01:01	No	No published evidence currently available.	—
HLA-DRB1*11:103	No	No published evidence currently available.	—
HLA-DRB1*13:41	No	No published evidence currently available.	—

## Discussion

A long-sustained adaptive immunity against pathogens is essential in preventing the host from re-infection with the same pathogen. With a high global prevalence of SARS-CoV-2 exposure, herein, we have demonstrated a long-term impact of COVID-19 on the immunity of hosts who have recovered from either severe or non-severe disease. Furthermore, our findings provide insight into the role of HLA polymorphisms in modulating COVID-19 severity among African patients, a population that remains underrepresented in global immunogenetic studies.

A fifth of C-19RPs had mild COVID-19-like symptoms at enrolment, suggesting symptom persistence after recovery, as reported in several long-COVID-19 studies (20). Post- COVID-19 syndromes are associated with risks for multiple organ system disorders, including pulmonary and neurological (21). Thus, future studies should monitor the long-term clinical effects of post- COVID-19 symptoms.

SARS-CoV-2 antibody titers during and post-infection depend on disease severity, with higher antibody levels detected in severe than non-severe recoverees (22, 23). Similarly, our data indicate higher infection-induced anti-SARS-CoV-2 spike IgG antibodies are detectable in severe than non-severe C-19RPs for up to 12 months post-infection. Importantly, our data further showed that all non-severe vaccinated C-19RPs had detectable and significantly higher SARS-CoV-2 antibody titers than unvaccinated C-19RPs, whereas no difference was observed between vaccinated and unvaccinated severe C-19RPs. Studies have shown that vaccination significantly elevates antibody responses in individuals who have already been exposed to the virus and to a greater extent in people recovered from mild COVID-19 (22–24). Our results indicate that the level of immune protection after recovery depends on disease severity, with non-severe convalescents likely to benefit more from vaccination than those who recovered from severe COVID-19. This highlights the need to prioritize vaccination of uninfected individuals and those who have recovered from non-severe COVID-19 cases, especially when resources are limited.

Memory B cells are crucial for a durable humoral immunity. Numerous studies have established a general persistence of memory B-cells after SARS-CoV-2 infection and/or vaccination (23, 25–27). We observed a non-significant trend towards a decreased fraction of switched memory B cells in the active infection and severe C-19RPs, as well as a significant decrease in naïve B cells in the actively infected group compared to the control group. These findings align with a study that showed severe COVID-19 mortality was associated with a decreased frequency of switched memory and naïve B cells (28). On the other hand, the frequencies of memory B cells were significantly higher in the active infection group than in the COVID-19 recovered participants, whereas the opposite was observed for naïve B cells. This indicates that during active infection, the frequencies of memory and naïve B cells are altered but are restored after recovery. However, the frequencies of the studied B-cell subsets did not correlate with the levels of virus-specific IgG in the blood. This suggests that despite the restoration of frequencies of B-cell subsets in COVID-19 recovered individuals, the absolute numbers and quality of these cells may remain compromised in some individuals. Indeed, persistence of exhausted B-cell populations post-COVID-19 infection has been reported (29).

We showed that the frequencies of CD4 but not CD8 T cell phenotypes are altered during active infections compared to the control group; however, these alterations are restored to homeostasis after disease recovery in both severe and non-severe patients. On the other hand, the notable decrease in the proportion of CD4 and CD8 central memory cells and the increase in effector memory cells in the C-19RPs compared to the active infection group aligns with findings from a longitudinal study that analyzed these cells at baseline and 6–8 months after recovery (30). In addition, studies have shown that T cell responses to SARS-CoV-2 upon re-stimulation with viral peptides may persist for over 12 months post-infection (31). Analysis of these responses, conducted for up to 12 months post-infection in our study, revealed no statistically significant differences between the groups and the controls, likely due to the small sample size of the control group. Nonetheless, the degranulation capacity of CD8 T cells was significantly higher in the severe group compared to the active infection group, indicating an improved response after recovering from severe disease. Interestingly, the CD8 T cell responses to a superantigen (SEB), measured by degranulation and cytokine production (IFNγ and TNFα), were enhanced, and again, only in the severe C-19RPs compared to the control, but when compared to the active infection, the degranulation capacity was enhanced in both the C-19RPs. In contrast, within the CD4 T cell compartment, the enhancement of responses was limited to degranulation and was observed in the severe C-19RPs only.

Structure-based computational models have identified a highly conserved sequence motif on the SARS-CoV-2 spike protein with a sequence and structure similar to a region on the SEB superantigen (32–34). This may imply that the elevated SEB responses observed in the severe C-19RPs were due to a preferential expression of host immunogenetic factors that bind both the SARS-CoV-2 spike protein and SEB motifs, causing a superantigenic response. Both superantigens and SARS-CoV-2 infection have been associated with the development of autoimmune or chronic inflammatory diseases (35–42). Therefore, the enhanced T cell responses to SEB among severe C-19RPs may predict the risk of future autoimmune diseases in this group.

We also showed that the CD4 and CD8 TNFα responses to an unrelated virus antigen (cytomegalovirus) were significantly higher compared to the active infection group, suggesting that during COVID-19 active infection, responses to other viruses are impaired but restored upon recovery. Our findings on T cell responses demonstrate important short-term immunological effects of COVID-19 and long-term T-cell hyperresponsiveness that SARS-CoV-2 leaves behind after the infection is cleared, as observed in patients recovered from severe COVID-19. Further *in vivo* animal experiments may confirm whether these enhanced responses can improve control of other unrelated infections, such as bacterial infections, in severely COVID-19-recovered hosts.

Studies on cytokine profiles in the serum of COVID-19 patients show elevated inflammatory cytokines, which are associated with the severity of the disease (43–45). Our study showed a non-significant trend toward increased serum cytokine levels in the actively infected group, likely due to the small sample size of the control group. Additionally, significantly higher levels of TNFα, IL8, MMP-8, IFNγ, and MPO in the active infection group compared to both severe and non-severe C-19RPs, and MMP-1 only in the severe C-19RPs, suggest that serum cytokine levels in COVID-19 patients, regardless of disease severity, return to homeostasis at similar kinetics after clearance of the infection.

The CD56Dim NK population is comprised of subsets of varying cytotoxic capacity, with CD56Dim_CD16Bright cell population being the most abundant cytotoxic NK cell subset in peripheral blood, with a relatively high degranulation capacity upon activation (11). The frequencies of CD56Dim_CD16Bright NK cells in our study were significantly elevated in severe and non-severe C-19RPs. Elevated frequencies of this NK subset have been associated with a strong potential for producing perforin, granzyme B, and IFNγ in vaccinated Omicron BA.2-infected patients (46). Although we were unable to determine which variant our participants were infected with, our findings suggest that SARS-CoV-2 infections typically maintain these activated NK cell subsets after recovery from both severe and non-severe forms of the disease. The frequencies of CD56Bright_CD16Dim NK cells were significantly higher in the active infection group than in the severe and non-severe C-19RPs. Studies have shown that this population of cells produces large amounts of cytokines upon monocyte activation (47, 48), which matches our findings that this population expanded significantly in the active infection group compared to the C-19RPs.

The expression of mRNA for Angiotensin-Converting Enzyme type 2 (ACE-2) receptor (entry receptor for SARS-CoV-2) has been shown to be higher in classical monocyte subsets compared to intermediate and non-classical monocytes (49). On the other hand, non-classical monocytes are reported to express higher levels of FcR-3A (FcR-mediated phagocytosis) gene than the other two subsets (50). These two properties were expected to alter the frequencies of the monocyte subsets in the active infection group or recovered participants, but our data suggest that monocyte subsets are not affected by COVID-19 during active infection or after recovery. Furthermore, two studies have shown that intermediate and non-classical monocytes were altered in convalescent COVID-19 patients; however, the extent of these alterations was inconsistent between the two studies (51, 52). The discrepancy between our findings and those of others may be attributed to factors such as vaccination status, virus strain, or sample size.

Consistent with the allele distributions observed in our study, the presence of several previously reported HLA risk alleles in severe cases suggests that host immunogenetic variation may influence COVID-19 disease outcomes in this population. Several alleles previously associated with severe disease in other populations, including HLA-A*03:01:01, HLA-B*08:01:01, HLA-DQB1*03:01:01 and HLA-DRB1*12:22, were observed exclusively in severe C-19RPs. These findings are consistent with studies conducted in European and other non-African populations that have reported associations between these alleles and increased susceptibility or more severe clinical outcomes (13, 14). These alleles in our study also suggest that certain immunogenetic mechanisms influencing SARS-CoV-2 pathogenesis may be shared across diverse ancestral backgrounds.

At the same time, several alleles associated with milder disease outcomes, such as HLA-A*02:02, HLA-C*05:01:01, HLA-DPB1*17:01 and HLA-C*08:02:01, were found only in non-severe C-19RPs. These alleles have previously been linked to reduced susceptibility or enhanced immune clearance, possibly through more efficient peptide presentation or NK cell-mediated cytotoxicity (16–19). Although our sample size was limited, the enrichment of these alleles in Tanzanian participants with mild COVID-19 suggests a plausible protective mechanism that may involve both innate and adaptive immune pathways.

Notably, several alleles found exclusively in either severity group have not yet been reported in the context of COVID-19. While their clinical significance remains to be established, the detection of these potentially novel associations highlights the value of studying African populations, which harbor some of the highest levels of HLA genetic diversity globally. Previous studies have shown that sub-Saharan African populations exhibit extensive HLA polymorphism and unique haplotypes that are underrepresented in global genomic datasets (53–55). Our findings, therefore, reinforce the importance of including African cohorts in immunogenetic studies of infectious diseases, as they may reveal population-specific immune signatures that remain undetected in predominantly Eurocentric studies (56). Larger studies integrating HLA genotyping with clinical, virological, and immunological data will be required to validate these candidate associations.

Our study has several limitations. Our major limitation was a small sample size, especially in the control group, due to a high COVID-19 seroprevalence in the region during the studied period. In addition, because vaccination campaigns in Tanzania started late (in July 2021), the number of vaccinated participants in this study, particularly among the severe recovered participants, was small, limiting the analysis of immune cell phenotyping and function by vaccination status and comorbidities. Furthermore, the sample size for HLA sequencing was modest due to limited resources; therefore, the results should be considered preliminary and hypothesis-generating. Although the exclusive presence of certain alleles in severe or non-severe cases is intriguing, the results should not be interpreted as causal associations, but rather as candidate signals warranting investigation in larger cohorts. Another limitation is that we did not assess HLA haplotypes or linkage disequilibrium patterns, which could influence associations. Also, functional assays to validate immunogenicity, peptide binding affinity, or downstream T-cell responses were not performed. Lastly, our findings may not be generalizable across the continent, as allele frequencies vary widely among African populations.

## Conclusions

We have shown that COVID-19 vaccination was particularly beneficial for non-severe C-19RPs, highlighting the benefits of vaccination in this group. These findings may be necessary in decision-making when vaccinating populations, especially in cases where resources are limited. Frequencies of B and NK cell subsets were altered in the C-19RPs, while CD4 T-cell subset alterations were only in the actively infected patients. The enhanced T-cell response to SEB in the severe C-19RPs suggests potential long-term T-cell hyperresponsiveness, warranting further research.

**Figure 7:**
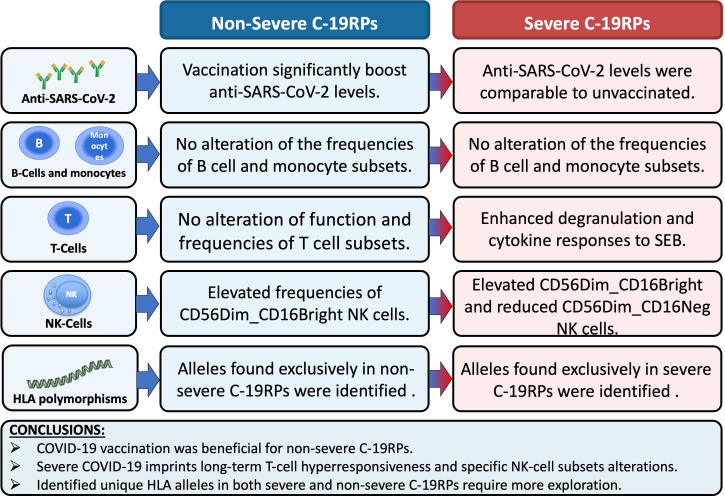
A schematic summary of the main study findings and conclusions. The antibody and nucleic acid icons were adapted from Servier Medical Art (https://smart.servier.com/), licensed under CC BY 4.0 (https://creativecommons.org/licenses/by/4.0/). .

Our study also identified both previously reported and potentially novel HLA alleles associated with COVID-19 severity in a Tanzanian cohort. Although our HLA results are more exploratory, they highlight the importance of integrating African genomic diversity into global immunogenetic studies. This may provide preliminary signals that can guide future large-scale investigations of host susceptibility and/or response to SARS-CoV-2. Given the limitations of current literature and database coverage, especially for African populations, these novel associations should be interpreted with caution until they are validated. Further studies with larger sample sizes, longitudinal follow-up, and functional validation will be crucial to confirm these associations and guide precision medicine approaches in Africa (a schematic representation summarizing the overall study findings is shown in **Figure 7**).

## Data Availability

The genetic data presented in the study are deposited in the NCBI repository, accession number PRJNA1438645. The rest of the raw data supporting the conclusions of this article will be made available by the authors on request.
