# Supplementary material for: Long-term immune profiling of COVID-19 recovered patients: effects of disease severity and vaccination
Source: Front Immunol. 2026 Apr 10;17:1699992. doi: 10.3389/fimmu.2026.1699992 (PMC13106164; doi:10.3389/fimmu.2026.1699992)
Supplement: Supplementary file 1 [file Supplementaryfile1.docx]

**Table S1A:** List of antibody panels used for staining the cells for flow cytometry analysis

**Table S1B:** List of SARS-CoV-2 nucleocapsid peptide sequences used in the peptide pool for stimulation.

| S/N | WUHAN | DELTA | OMICRON |
| --- | --- | --- | --- |
| 1 | TFGGPSDSTGSNQNGER | TFGGPSDSTGSNQNGER | TFGGPSDSTGSNQNG-- |
| 2 | DSTGSNQNGERSGAR | DSTGSNQNGERSGAR | DSTGSNQNG---GAR |
| 3 | SNQNGERSGARSKQR | SNQNGERSGARSKQR | SNQNG---GARSKQR |
| 4 | GERSGARSKQRR | GERSGARSKQRR | G---GARSKQRR |
| 5 | ERSGARSKQRRPQGL | ERSGARSKQRRPQGL | ---GARSKQRRPQGL |
| 6 | SWFTALTQHGKEDLK | SWFTALTQHGKEGLK | SWFTALTQHGKEDLK |
| 7 | ALTQHGKEDLKFPR | ALTQHGKEGLKFPR | ALTQHGKEDLKFPR |
| 8 | TQHGKEDLKFPR | TQHGKEGLKFPR | TQHGKEDLKFPR |
| 9 | QHGKEDLKFPRG | QHGKEGLKFPRG | QHGKEDLKFPRG |
| 10 | HGKEDLKFPRGQGV | HGKEGLKFPRGQG | HGKEDLKFPRGQGV |
| 11 | EDLKFPRGQGVPI | EGLKFPRGQGVPI | EDLKFPRGQGVPI |
| 12 | SSRNSTPGSSRGTSPA | SSRNSTPGSSMGTSPA | SSRNSTPGSSKRTSPA |
| 13 | TPGSSRGTSPARMA | TPGSSMGTSPARMA | TPGSSKRTSPARMA |
| 14 | SSRGTSPARMAGNGGDA | SSMGTSPARMAGNGCDA | SSKRTSPARMAGNGGDA |
| 15 | TEPKKDKKKKADETQA | TEPKKDKKKKAYETQA | TEPKKDKKKKADETQA |
| 16 | DKKKKADETQALPQR | DKKKKAYETQALPQR | DKKKKADETQALPQR |
| 17 | KADETQALPQRQKK | KAYETQALPQRQKK | KADETQALPQRQKK |

**Table S2**: Participants’ comorbidities

| **Variable** | **Non-Severe n (%)** | **Severe n (%)** | **Active n (%)** |
| --- | --- | --- | --- |
| Hypertension | 11 (13.3) | 4 (22.2) | 9 (50.0) |
| Diabetes Mellitus type II | 5 (6.0) | 2 (11.1) | 6 (33.3) |
| HIV | 1 (1.2) | 1 (5.6) | 2 (11.1) |
| Tuberculosis |  |  | 1 (5.6) |
| Asthma | 2 (2.4) |  | 1 (5.6) |
| Cardiac diseases | 1 (1.2) |  | 1 (5.6) |
| Chronic Kidney Disease |  |  | 3 (16.7) |
| Malignant | 1 (1.2) |  |  |
| Others* | 5 (6.0) | 2 (11.1) | 2 (11.1) |

*Include: Spondylosis, Peptic ulcer disease, Stroke, Gout and Glaucoma


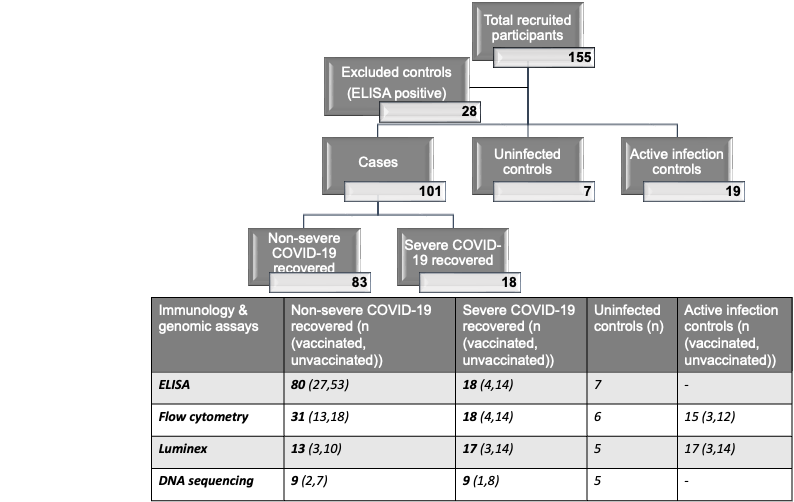


**Figure S1:** Number of recruited participants, grouping, and selected number of participant samples for individual immunoassays and sequencing.


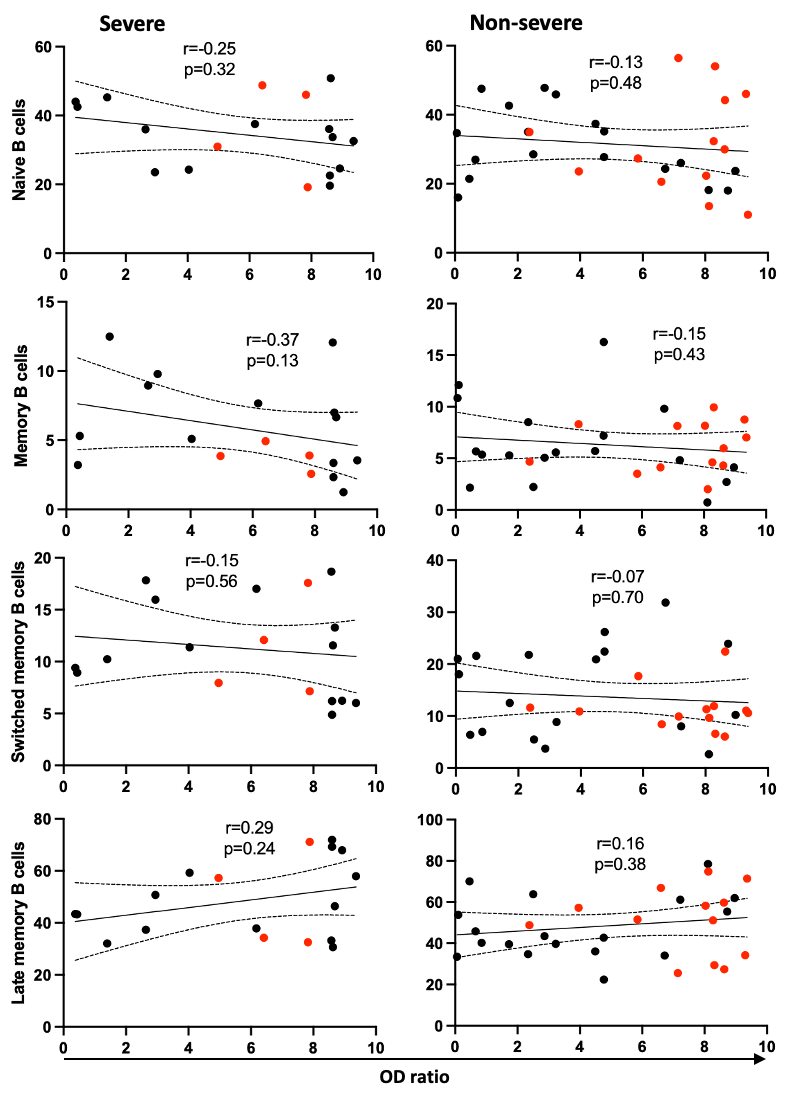


**Figure S2:** **Frequencies of B cell subsets and the antibody titers (OD ratio).** Correlation between the frequencies of differentiated B cell subsets (naïve, memory, switched memory, and late memory B cells) and the OD ratio in vaccinated (n=4; red dots) and unvaccinated (n= 14; black dots) severe (left panels) and non-severe (n=13; vaccinated (red dots), n=18 unvaccinated (black dots) (right panels)). Statistical analysis was done using Spearman’s rank correlation.


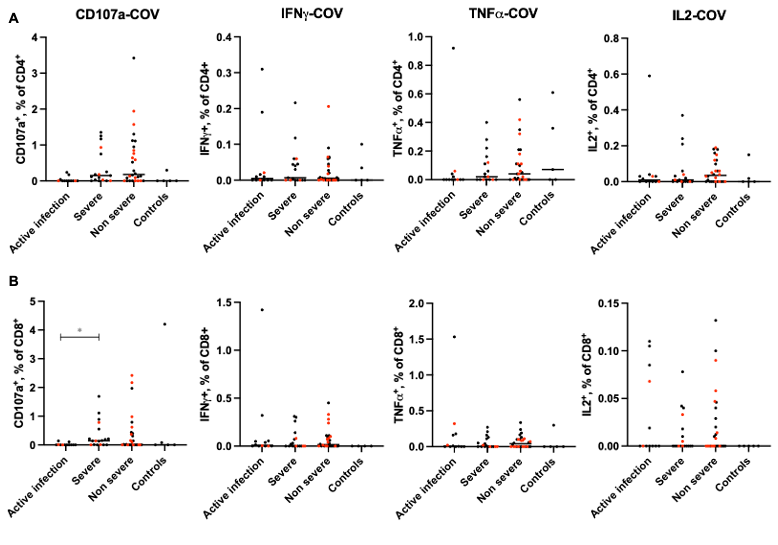


**Figure S3:** **Effects of COVID-19 on the T cell responses to SARS-CoV-2 antigen stimulation**. The frequencies of CD4 (A) and CD8 (B) T cell responses to SARS-CoV-2 nucleocapsid peptide pools, measured by degranulation (CD107a) or cytokine production (interferon gamma (IFN) or Tumor Necrosis Factor (TNF)), were compared between the infected and control groups. Each dot represents a participant (red: vaccinated, black: unvaccinated), and the horizontal line represents the group median. ^*^p<0.05 (Kruskal-Wallis test followed by Dunn’s post-analysis).


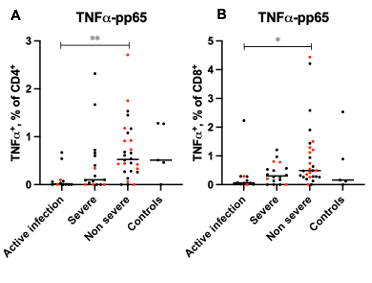


**Figure S4:** **Effects of COVID-19 on the T cell responses to CMV peptides (pp65) stimulation**. The frequencies of CD4 (A) and CD8 (B) T cell responses to pp65, measured by TNFα cytokine production, were compared between the infected and control groups. Each dot represents a participant (red: vaccinated, black: unvaccinated), and the horizontal line represents the median of the group. ^*^p<0.05; ^**^p<0.01 (Kruskal-Wallis test followed by Dunn’s post-analysis).


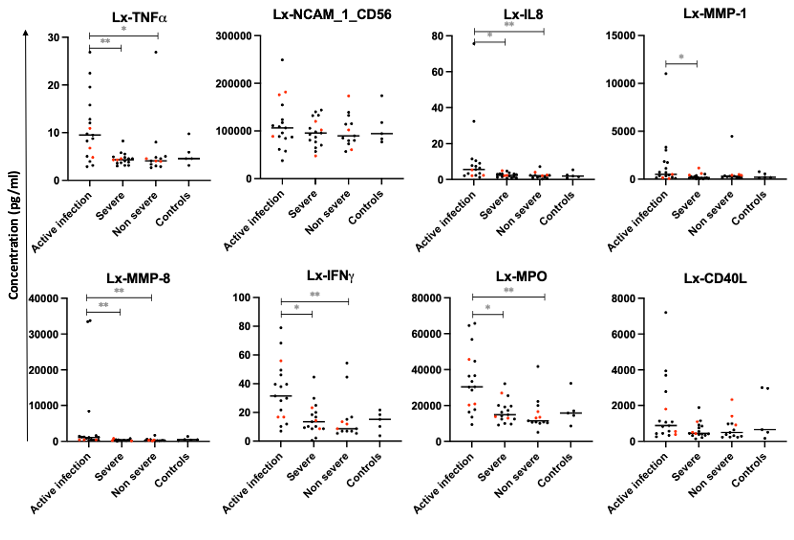


**Figure S5:** **Effects of COVID-19 on the serum cytokine levels**. The concentration of different cytokines in the serum measured by LUMINEX assay was compared between the infected and control groups. Each dot represents a participant (red: vaccinated, black: unvaccinated), and the horizontal line represents the group median. ^*^p<0.05; ^**^p<0.01 (Kruskal-Wallis test followed by Dunn’s post-analysis).

**
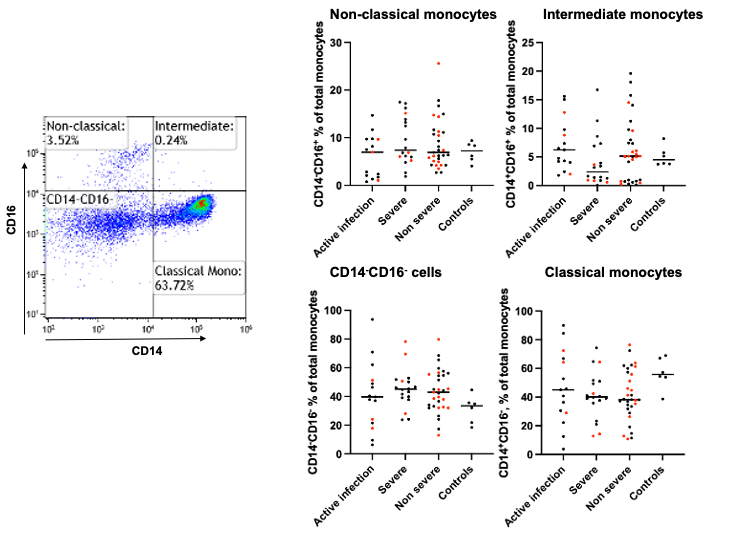
Figure S6: Effects of COVID-19 on the monocyte subsets**. The frequencies of the three monocyte subsets in the peripheral blood of actively infected, severe, and non-severe C-19RPs were compared to uninfected control participants. No difference was found between the infected and control groups in any of the three monocyte subsets. Each dot represents a participant (red: vaccinated, black: unvaccinated), and the horizontal line represents the median of the group. Statistical analysis was done using the Kruskal-Wallis test, followed by Dunn’s post-analysis
